# The Decrease in Serum sRAGE Levels Upon Smoking is Associated with Activated Neutrophils

**DOI:** 10.1007/s00408-022-00585-4

**Published:** 2022-10-25

**Authors:** Valerie R. Wiersma, Susan J. M. Hoonhorst, Nick H. T. ten Hacken, Maarten van den Berge, Dirk-Jan Slebos, Simon D. Pouwels

**Affiliations:** 1 Department of Hematology, Cancer Research Center Groningen, University Medical Center Groningen, Groningen, The Netherlands; 2 Department of Pulmonary Diseases, University Medical Center Groningen, Hanzeplein 1, 9713 GZ Groningen, The Netherlands; 3 Groningen Research Institute for Asthma and COPD (GRIAC), University Medical Center Groningen, Hanzeplein 1, 9713 GZ Groningen, The Netherlands; 4 Department of Pathology and Medical Biology, University Medical Center Groningen, Hanzeplein 1, 9713 GZ Groningen, The Netherlands

**Keywords:** COPD, Biomarkers, sRAGE, Cigarette smoke, CD11b

## Abstract

The serum level of the soluble Receptor for Advanced Glycation End-products (sRAGE) is a promising blood biomarker for the development, severity, and progression of chronic obstructive pulmonary disease (COPD). However, cigarette smoking causes a nearly instant drop in circulating sRAGE levels, strongly impacting on the variability in sRAGE levels. In the current study, we investigated the possible mechanism behind the sudden drop in sRAGE upon smoking. We showed that the number of activated neutrophils in blood significantly increases within two hours upon smoking three cigarettes within one hour. Furthermore, an increased expression of the leukocyte activation marker CD11b, which is a known ligand for RAGE, was observed upon smoking. Additionally, the *in vitro* activation of neutrophils increased their capacity to bind sRAGE. Together, these data indicate that smoking activates neutrophils in the circulation with concomitant upregulation of the RAGE ligand CD11b, leading to reduced levels of sRAGE in serum.

## Introduction

Chronic obstructive pulmonary disease (COPD), is a severe lung disease characterized by chronic, mostly neutrophilic, inflammation of the lower airways (chronic bronchitis), as well as progressive destruction of the alveoli (emphysema). To date, one of the best blood biomarkers for the development, severity, and progression of COPD is the soluble receptor for advanced glycation end-products (sRAGE) [1, 2]. sRAGE is released into the bloodstream upon cleavage of the full-length pro-inflammatory pattern recognition receptor RAGE by proteases [3]. Additionally, a specific splice variant lacking the transmembrane and cytoplasmic domains, endogenous secretory (es)RAGE is directly secreted upon translation, adding to the circulating sRAGE pool [4]. Over the past years, it has been consistently shown that the levels of sRAGE are lower in COPD patients compared to healthy controls [2, 5, 6]. This was confirmed by a meta-analysis combining serum sRAGE data from multiple large cohorts [7]. Moreover, serum sRAGE levels are more associated with emphysema, especially centrilobular emphysema, compared to chronic bronchitis [6, 8]. Additionally, a genetic variant was identified (rs2070600) that significantly affects circulating levels of sRAGE [6, 9, 10]. In contrast, circulating sRAGE levels are not significantly affected by basic patient characteristics, such as age, sex, or body mass index [6, 7, 10]. Recently, we showed a strong decrease in the serum levels of sRAGE within 2 h upon smoking [11]. The mechanism of the cigarette smoke-induced decrease in blood sRAGE is currently unknown. As the decrease in serum sRAGE levels upon smoking occurs nearly instantly, sRAGE is likely to be captured and bound upon smoking. Previously, it has been described that RAGE can bind to the i-domain of CD11b [12], an important activation marker of several leukocytes, including neutrophils [13]. Binding of RAGE to CD11b within the Macrophage-1 antigen (Mac-1) complement receptor induces a pro-inflammatory response and the recruitment of leucocytes [14]. In the current study, we hypothesized that cigarette smoking increases the number of CD11b^+^ activated neutrophils in blood, which can bind sRAGE, lowering the measurable levels in serum.

## Methods

In order to assess the effect of smoking on neutrophil counts and their activation status, blood was collected from 77 young intermittent smokers without airway obstruction [15]. The subjects were between 21 and 62 years of age, 66% was male and all subjects had normal lung function as defined by a forced expiratory volume in 1 s divided by the forced vital capacity (FEV_1_/FVC) of over 75% [15]. Blood samples were collected after at least two days without smoking (before smoking) and two hours after smoking three cigarettes within one hour (after smoking). The study was approved by the medical ethics committee of the university medical center Groningen (UMCG), and all participants provided written informed consent (Clinicaltrials.gov Identifier: NCT00807469). Furthermore, all clinical procedures were performed according to the standards set by the latest Declaration of Helsinki. The number of neutrophils and CD11b^+^ leukocytes in patient blood and the amount of RAGE bound to neutrophils in *in vitro* assays were determined with flow cytometry as described before [15]. In short, before and after smoking, peripheral blood was collected in tubes containing sodium heparin. Red blood cells were lysed and washed with 5 mL cold PBS + 10 g/L albumin + 0.32% sodium citrate. Cells were primed for 5 min at 37 °C before being stained for 1 h at 4 °C with antibodies against Mac-1 (CD11b, clone 2LPM19c, DAKO, Copenhagen, Denmark), L-selectin (CD62L, clone DREG56, BD Pharmingen, San Diego, CA, USA), and RAGE (MAB11451, clone 176,902, R&D Systems, Abingdon, U.K.). Neutrophils were classified as CD62L^high^ expressing granulocytes, the latter being selected based on forward and sideward scatter characteristics. sRAGE levels in serum and *in vitro* assays were measured using ELISA (R&D Systems, DY1145, Minneapolis, MN, USA).

## Results

**Fig. 1:**
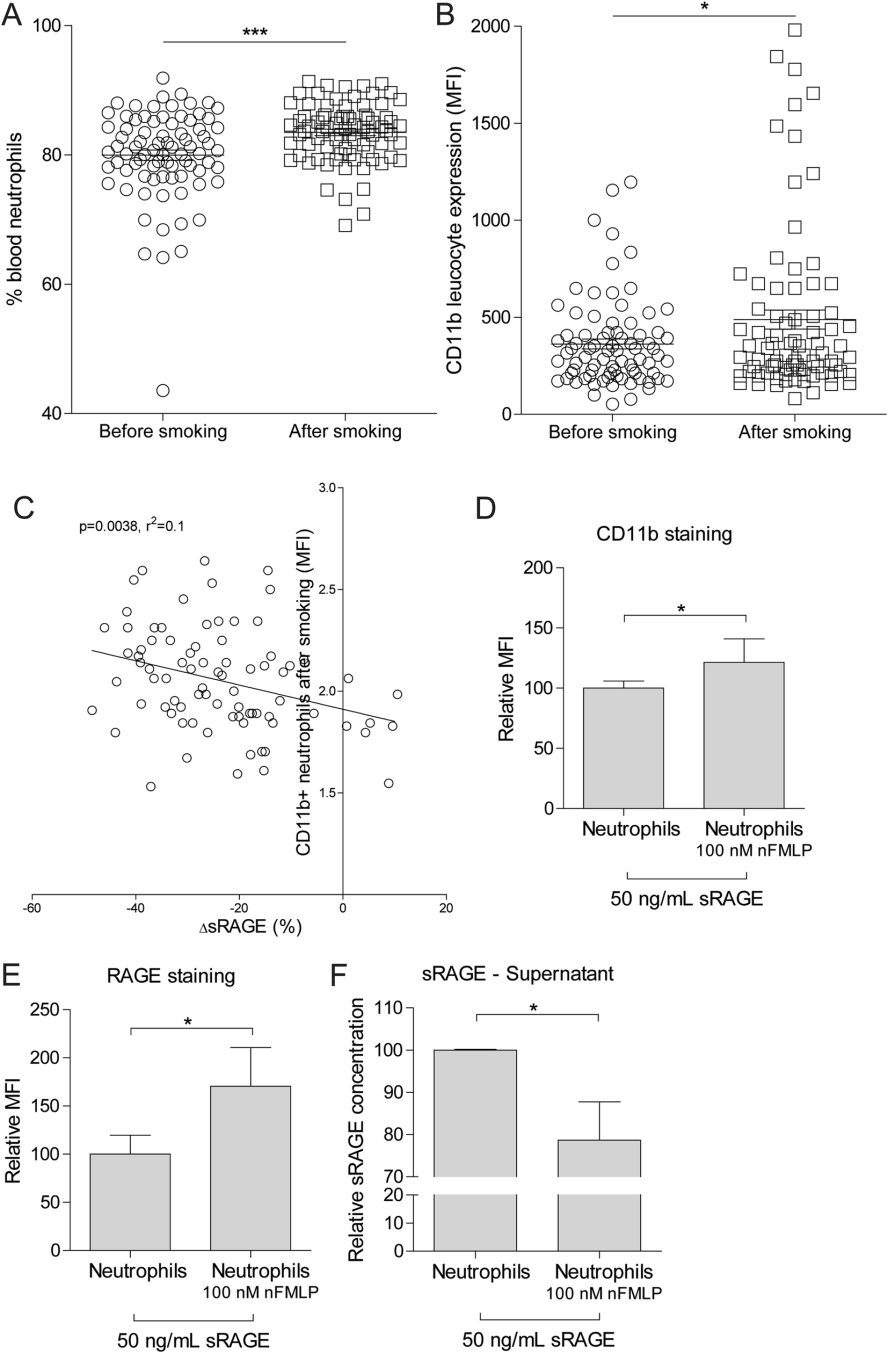
Activated neutrophils display increased surface binding of sRAGE. **A** The number of neutrophils was measured in blood of 77 intermittent smokers without airway obstruction. Blood was collected after at least two days without smoking (before smoking) and two hours after smoking three cigarettes within one hour (after smoking). The percentage of neutrophils was determined by flow cytometry. The granulocyte population was 7 isolated using forward and sideward scatter characteristics and the neutrophil population was characterized as CD62Lhigh expressing granulocytes. **B** CD11b expression was assessed on blood leucocytes before and after smoking using flow cytometry. **C** The association between the percentage decrease in serum sRAGE levels before and after smoking with the expression of CD11b on neutrophils after smoking. **D**–**E** Neutrophils were isolated from healthy controls (*n* = 5) and incubated for one hour in RPMI with 10% fetal calf serum and 50 ng/mL recombinant sRAGE. The effect of activation by 100 nM N-Formylmethionyl-leucyl-phenylalanine (fMLP) for hour on the surface expression of CD11b and RAGE was assessed by flow cytometry. **F** The residual levels of sRAGE after activation with 100 nM fMLP for one hour were assessed in cell-free supernatant using ELISA. Data are shown as individual data points or mean ± SEM. Statistical significance was assessed using a Mann–Whitney U test to compare conditions or with a linear regression analysis to assess associations, * *p* < 0.05, *** *P* < 0.001

Here, it was found that smoking three cigarettes acutely increased the number of neutrophils and the number of CD11b^+^ leukocytes in blood (Fig. 1A–B). Additionally, a more pronounced decrease in sRAGE levels upon smoking was associated with a higher expression of CD11b on neutrophils in blood (Fig. 1C). Next, neutrophils were isolated from healthy controls and *in vitro* incubated in RPMI cell culture medium containing 50 ng/mL recombinant sRAGE (Merck, SRP6051, Kenilworth, New Jersey). Activation of the neutrophils by stimulation with 100 nM N-Formylmethionyl-leucyl-phenylalanine (fMLP) for one hour increased the surface expression of CD11b as well as the amount of sRAGE which was bound to the neutrophils (Fig. 1D–E). Of note, activation of neutrophils in the absence of recombinant sRAGE did not result in any surface expression of RAGE, indicating that RAGE is not noteworthy expressed on the surface of neutrophils (*data not shown*). To further confirm that activated neutrophils bind sRAGE, the levels of sRAGE in supernatant of non-activated and fMLP-activated neutrophils were measured. Here, a significant decrease in residual sRAGE levels was observed in the supernatant of activated neutrophils (Fig. 1F).

## Discussion

This study contributes to the knowledge that cigarette smoking activates neutrophils and that these activated neutrophils can bind sRAGE. Previously, it was shown that smoking acutely decreases the serum levels of the COPD biomarker sRAGE up to 50% [11]. This finding has strong implications for the effectiveness and usability of sRAGE as COPD biomarker, as this may induce large variations in sRAGE values based upon the smoking habits of the subjects [16–18]. Therefore, it is important to understand the mechanism behind the cigarette smoke-induced drop in serum sRAGE levels. The current study shows that the number of activated neutrophils increases in blood within two hours after smoking three cigarettes. Additionally, using *in vitro* experiments, we showed that activated neutrophils are capable of binding sRAGE on their surface and reducing the levels of sRAGE from the surrounding fluid. These data indicate that activated neutrophils can be responsible for the cigarette-smoke-induced drop in serum sRAGE levels. Furthermore, the binding of sRAGE to activated neutrophils may also contribute to the widely observed decrease in serum sRAGE levels in COPD patients, a predominantly neutrophilic inflammatory disease. Future studies should investigate whether also other surface-markers of activated neutrophils, besides the known RAGE ligand, CD11b [13], are capable of binding sRAGE. Together, this study indicates that refraining from smoking for at least a day prior to blood sampling may decrease the variability and increase the strength of sRAGE as a COPD biomarker.
